# Chronic Granulomatous Invasive Fungal Rhinosinusitis With Intracranial Extension in an Immunocompetent Patient: A Case Report

**DOI:** 10.7759/cureus.71236

**Published:** 2024-10-10

**Authors:** Sylvia M Mosito, Shehzad Saeedullah, Liam Robinson, Samuel J Houghton, Christian Quitter

**Affiliations:** 1 Otolaryngology - Head and Neck Surgery, University of Pretoria, Pretoria, ZAF; 2 Otolaryngology - Head and Neck Surgery, Northwest General Hospital, Peshawar, PAK; 3 Maxillofacial Radiology, Steve Biko Academic Hospital, Pretoria, ZAF; 4 Otorhinolaryngology, Steve Biko Academic Hospital, Pretoria, ZAF; 5 Otorhinolaryngology, University of Pretoria, Pretoria, ZAF

**Keywords:** cgifrs, immunocompetent, intracranial extension, itraconazole, liposomal amphotericin, sinonasal malignancy

## Abstract

Chronic granulomatous invasive fungal rhinosinusitis (CGIFRS) is an uncommon disease pathology seen in immunocompetent patients. The most common causative fungal agents reported in the literature are members of the Aspergillus species. CGIFRS may be mistaken for sinonasal malignancy because of its invasive pattern. This article reports a case of CGIFRS in an immunocompetent male patient with intracranial extension who responded well to antifungals (liposomal Amphotericin and Itraconazole) after surgical debulking. Appropriate clinicopathological evaluation and diagnosis are essential for proper management. Only a few such cases have been reported in the literature. In the Republic of South Africa, no case of CGIFRS with intracranial extension has been reported prior to this case.

## Introduction

Fungal rhinosinusitis (FRS) is a condition characterized by inflammation of the mucosal lining in the nasal cavity and paranasal sinuses due to the presence of fungus. FRS is a rare disease entity that has traditionally been categorized into invasive and non-invasive types. The invasive type is marked by the invasion of fungus into the surrounding tissues through the superficial mucosa while the non-invasive type lacks this feature. If left untreated, the invasive type can spread to the eyes, brain, and other surrounding tissues, leading to the destruction of tissues, vision impairment, and death. Invasive FRS has been further classified into acute fulminant FRS, which typically presents in immunocompromised patients (e.g. HIV positive, diabetics, and organ transplant), patients receiving chemotherapeutic drugs, chronic invasive fungal rhinosinusitis (CIFRS), and chronic granulomatous invasive fungal rhinosinusitis (CGIFRS), which commonly occur in immunocompetent patients. Non-invasive FRS includes fungal mycetoma, saprophytic fungal infestation, and allergic FRS [1].

Chronic granulomatous invasive fungal rhinosinusitis (CGIFRS) is characterized by fungal invasion into the underlying tissues of the nasal cavity and paranasal sinuses in conjunction with the presence of immune cell aggregates (granulomas) [2]. The disease has a long course of progression, which can lead to a delay in diagnosis and appropriate management. Patients frequently present with nonspecific symptoms, including nasal obstruction, mucopurulent nasal discharge, epistaxis (nosebleed), headache, and facial pain. Thus, a high index of suspicion is required for diagnosis. Untreated CGIFRS has the potential to invade surrounding structures (e.g. intracranial cavity and orbit) [3]. If the orbit is involved, symptoms may include blurred vision, progressive visual loss, chemosis (conjunctival edema), and proptosis (bulging of the eyes) [4]. Although active infection with Aspergillus is unlikely in immunocompetent patients, Aspergillus is the most commonly cultured fungus in immunocompetent patients; hence, Aspergillosis should be suspected in individuals with refractory or recurrent sinusitis [1,5].

It is important to note that CGIFRS may sometimes be indistinguishable from sinonasal malignancy on imaging; therefore, biopsy is crucial to confirm the diagnosis [6]. The histology should be specific as to whether there is mucosal invasion or not. Delayed diagnosis and treatment of CGIFRS may lead to poor therapeutic outcomes and death [2]. Management of invasive fungal rhinosinusitis is still controversial and depends on multiple factors such as the host's immunity and degree of tissue invasion. The recommended treatment is surgical debridement of the abnormal tissue and anti-fungal therapy to target the infected area [7]. This case report will assist in the early diagnosis and management of CGIFRS to prevent morbidity and mortality.

## Case presentation

We present a case report of a 32-year-old male patient who presented at our institution with an eight-month history of nasal symptoms: persistent bilateral nasal obstruction (more pronounced on the left than the right), hyposmia, mucopurulent nasal discharge, and intermittent episodes of epistaxis. Initially, the patient experienced hyposmia, followed three months later by mucopurulent nasal discharge, nasal obstruction, and epistaxis. Upon further inquiry, the patient displayed symptoms of allergic rhinitis. He had undergone two endoscopic sinus procedures in 2009 and 2012 for chronic rhinosinusitis with nasal polyposis at a private institution; however, histopathological reports were not available. The patient had applied topical intranasal corticosteroids (fluticasone furoate) intermittently for more than eight years. The patient admitted to non-compliance with medication and did not attend follow-up consultations with the treating institution.

Upon clinical examination, the patient displayed hypertelorism, proptosis, and a nasal hump. Anterior rhinoscopy confirmed bilateral nasal polyposis (more pronounced on the left than the right) and inflamed nasal mucosa. Blood serum tests and high-resolution CT scans of the paranasal sinuses and brain were performed for diagnostic workup. The total immunoglobulin E (IgE) was 374.0 IU/ml (normal range: 0.0-100.0 IU/ml), and specific antibodies to Alternaria alternata were elevated with a value of 3.31 IU/ml (normal range: 0.0-0.35 IU/ml). These findings indicated that the patient also suffered from allergic rhinitis as a result of the Alternaria alternata antigen.

**Figure 1:**
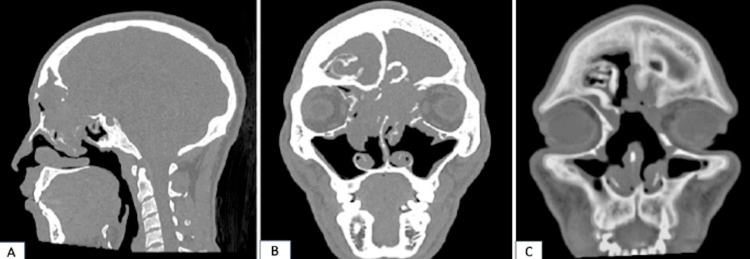
Preoperative and postoperative CT scans of the paranasal sinuses A. Sagittal view; B. Coronal view, soft tissue window displaying an ill-defined lesion anterior, posterior ethmoids, and frontal sinus causing bony erosion/remodeling and intracranial extension; C. Three months postoperative CT scan of the paranasal sinuses with a minimal soft tissue lesion seen in the left frontal sinus

A high-resolution computed tomography (HRCT) scan of the paranasal sinuses and brain revealed a destructive lesion in the anterior and posterior ethmoids, extending into the anterior skull base and eroding the medial wall of the left orbit, which correlates with the clinical finding of proptosis (Figure 1). A biopsy of the polyps was performed prior to surgery and yielded results consistent with cylindrical cell papilloma, a variant of sinonasal Schneiderian papilloma.

The patient underwent extended endoscopic sinonasal resection of the lesion. Intraoperative findings revealed a fibrotic lesion adherent to the dura and invading brain tissue. The tumor adherent to the brain parenchyma was left in situ to avoid morbid complications of intracranial bleeding and cognitive dysfunction. The expected complication of a CSF leak was managed intraoperatively. The defect was reconstructed with two layers of closure (abdominal fat and a sinonasal-free flap from the nasal floor) in conjunction with Tisseel glue (Baxter, Deerfield, Illinois, US). The invasion of the lesion into the nasoseptal flap resulted in its omission from the reconstruction.

**Figure 2:**
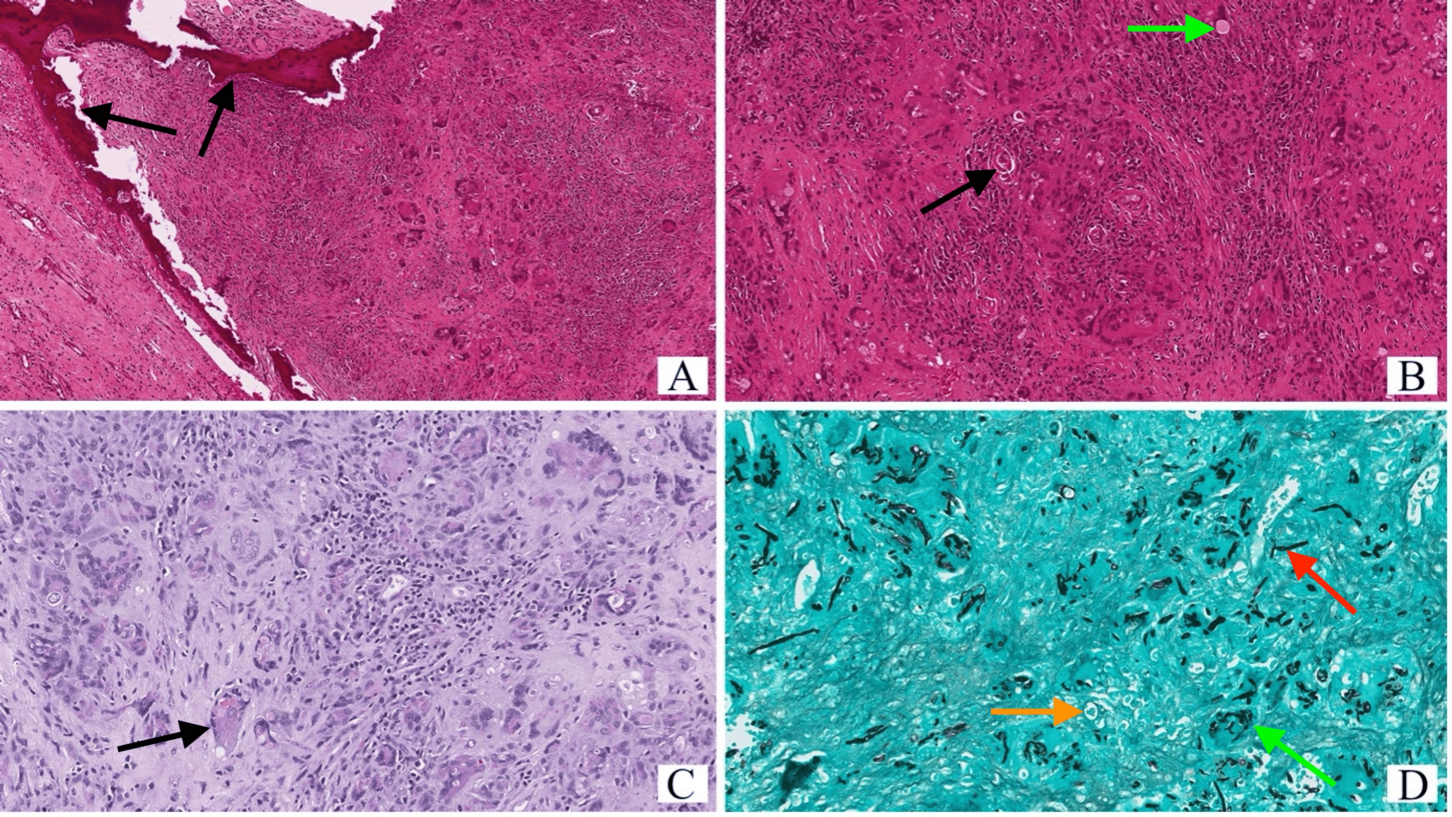
Histopathological findings A. H&E-stained section showing granulomatous inflammation with destruction of the adjacent bone (black arrows) (original magnification X 10). B. H&E-stained section showing vague epithelioid granulomas with abundant multinucleated giant cells (black arrow) and inconspicuous infective agents (green arrow) (original magnification X 40). C. Periodic acid-Schiff (PAS)-stained section vaguely highlighting the fungal elements (black arrow) (original magnification X 40). D. Grocott-Gomori histochemical stain showing mixed fungal agents consisting of septate hyphae (red arrow), pseudohyphae (green arrow), and yeasts (orange arrow) (original magnification X 40).

Histological examination revealed decomposition of the adjacent bone due to granulomatous inflammation. Additionally, epithelioid granulomas with numerous multinucleated cells were discovered in the presence of infective agents (Figure 2). Furthermore, mixed fungal agents (septate hyphae, pseudohyphae, and yeast) were identified (Figure 2). The final histological diagnosis displayed CGIFRS.

**Figure 3:**
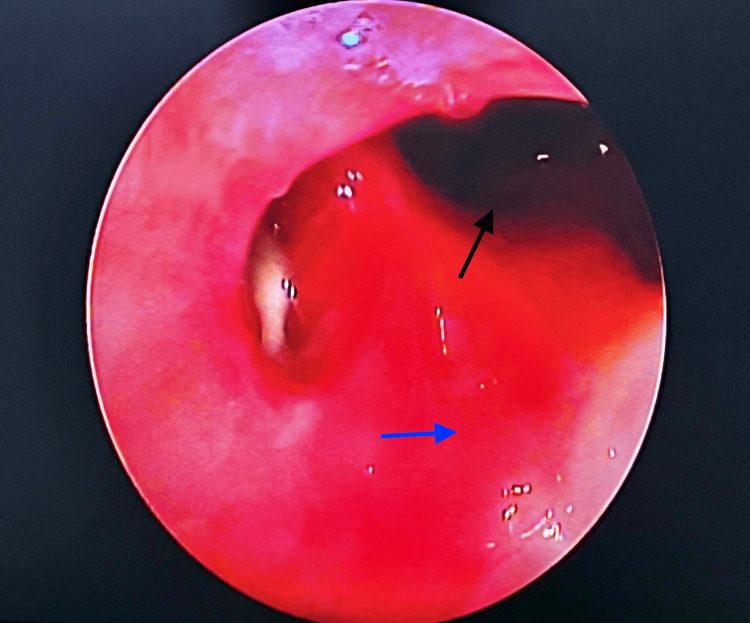
Postoperative endoscopic view of DRAF 3 procedure (three years following surgical excision) The black arrow indicates the frontal sinus cavity. The blue arrow indicates the reconstructed cribriform area.

The patient underwent a serial endoscopic examination on follow-up to rule out disease recurrence and frontal sinus recess stenosis (Figure 3).

Despite the patient's elevated IgE levels and specific antibodies to Alternaria alternata, the patient did not meet the diagnostic criteria for allergic FRS according to the Bent and Kuhn classification system [8]. Thus, it can be concluded that the patient was suffering from two separate disease entities: CGIFRS and allergic rhinitis. 

## Discussion

CGIFRS is a subtype of invasive FRS that typically occurs in immunocompetent patients presenting with symptoms lasting more than 12 weeks. The disease can also have an indolent course, with symptoms lasting from months to years [9]. Patients commonly present with nonspecific nasal symptoms such as nasal obstruction (unilateral more prevalent than bilateral), purulent or blood-stained nasal discharge, hyposmia/cacosmia, and a nasal mass. Occasionally, patients may present with atypical symptoms, including hypertelorism and proptosis, when the disease is extensive and causes bony expansion [9-11]. Headaches, seizures, cranial nerve fall-outs, or neurological deficits are symptoms and signs of intracranial extension [12].

A non-contrast CT scan of the paranasal sinuses is the preferred imaging modality for evaluating fungal sinusitis. It commonly demonstrates hyperdense calcified lesions in the affected sinuses and/or bony destruction or expansion of the adjacent sinuses [13]. The CT scan features may mimic sinonasal malignancy due to the destructive nature of the fungal infection [6]. An MRI scan is superior to a CT scan in assessing the extent of soft tissue, orbit, and brain involvement. The lesion may appear hypointense on T1 and markedly hypointense or show loss of signal void on T2 due to increased protein content [13,14].

Histologically, CGIFRS appears as fungal hyphae infiltrating the mucosa and submucosa, with surrounding chronic suppurative granulomatous inflammation, purulent inflammation, occasional necrosis, and invasion of the blood vessels by the fungus [15,16].

The current recommended management of CGIFRS is endoscopic surgical debridement followed by the use of antifungals (Itraconazole/Voriconazole) for a period of 6 to 18 months [17]. Following endoscopic debridement, our institution's preceding protocol was used. The patient in our case report received an intravenous course of Amphotericin B for two weeks, followed by oral Itraconazole for a period of three months. Nevertheless, recent literature has exhibited azoles to be more efficacious than Amphotericin B and indicates that Amphotericin B plays no role in the treatment of CGIFRS [5,17].

After three months of treatment, the patient continued to complain of mild headache and pain in the left forehead. A repeat CT scan of the paranasal sinuses showed a homogeneous lesion in the left frontal sinus. In view of these findings, the patient subsequently underwent revision endoscopic debridement of the left frontal sinus. Histopathological examination of the specimen showed no evidence of granulomas; however, features of fungal invasion to surrounding tissues remained. Following another three months of oral Itraconazole, the patient was asymptomatic. The patient received a total six-month course of oral Itraconazole. The patient was kept under close surveillance post-treatment to monitor for disease recurrence for three years, with biannual CT scans.

Cases of CGIFRS have been reported globally [2]; however, none have been reported in the Republic of South Africa. Due to the nature of the disease’s symptoms and signs, a high index of suspicion is required for early diagnosis of CGIFRS, as it can lead to significant morbidity and even mortality resulting from its aggressive nature. Surgical debridement and antifungals form the core of treatment. Intracranial extension of CGIFRS can mimic sinonasal malignancy on imaging and needs to be differentiated from it owing to entirely different treatment strategies. The prognostic outcome of CGIFRS in immunocompetent patients is favorable if treatment is initiated promptly. Since treatment for sinonasal malignancy with extensive intracranial invasion is palliative due to poor prognosis, histopathological diagnosis is a crucial tool needed to distinguish between the two. Patients with CGIFRS should be kept under close surveillance post-treatment to monitor for disease recurrence. 

## Conclusions

This case highlights the importance of early diagnosis and treatment in managing CGIFRS, particularly when intracranial extension is evident. Based on our findings and a literature review, we recommend a combination of endoscopic surgical debridement and antifungal therapy (Itraconazole or Voriconazole) for 6 to 18 months. Patients with CGIFRS should be monitored closely, and cases should be followed up closely to rule out disease recurrence. Due to the rarity of CGIFRS and the lack of data in the Republic of South Africa, a prospective multicenter study is justified in the future to establish management protocols for this rare disease spectrum.
